# Immunohistochemical analysis of changes in signaling pathway activation downstream of growth factor receptors in pancreatic duct cell carcinogenesis

**DOI:** 10.1186/1471-2407-8-43

**Published:** 2008-02-06

**Authors:** Nhu-An Pham, Joerg Schwock, Vladimir Iakovlev, Greg Pond, David W Hedley, Ming-Sound Tsao

**Affiliations:** 1 Department of Medical Biophysics, University of Toronto, Toronto, ON, Canada; 2 Department of Laboratory Medicine and Pathobiology, University of Toronto, Toronto, ON, Canada; 3 Department of Biostatistics, Princess Margaret Hospital, Toronto, ON, Canada; 4 Department of Medical Oncology, Princess Margaret Hospital, Toronto, ON, Canada; 5 Department of Pathology, University Health Network, Toronto, ON, Canada

## Abstract

**Background:**

The pathogenesis of pancreatic ductal adenocarcinoma (PDAC) involves multi-stage development of molecular aberrations affecting signaling pathways that regulate cancer growth and progression. This study was performed to gain a better understanding of the abnormal signaling that occurs in PDAC compared with normal duct epithelia.

**Methods:**

We performed immunohistochemistry on a tissue microarray of 26 PDAC, 13 normal appearing adjacent pancreatic ductal epithelia, and 12 normal non-PDAC ducts. We compared the levels of 18 signaling proteins including growth factor receptors, tumor suppressors and 13 of their putative downstream phosphorylated (p-) signal transducers in PDAC to those in normal ductal epithelia.

**Results:**

The overall profiles of signaling protein expression levels, activation states and sub-cellular distribution in PDAC cells were distinguishable from non-neoplastic ductal epithelia. The ERK pathway activation was correlated with high levels of ^S2448^p-mTOR (100%, p = 0.05), ^T389^p-S6K (100%, p = 0.02 and ^S235/236^p-S6 (86%, p = 0.005). Additionally, ^T389^p-S6K correlated with ^S727^p-STAT3 (86%, p = 0.005). Advanced tumors with lymph node metastasis were characterized by high levels of ^S276^p-NFκB (100%, p = 0.05) and ^S9^p-GSK3β (100%, p = 0.05). High levels of PKBβ/AKT2, EGFR, as well as nuclear ^T202/Y204^p-ERK and ^T180/Y182^p-p38 were observed in normal ducts adjacent to PDAC compared with non-cancerous pancreas.

**Conclusion:**

Multiple signaling proteins are activated in pancreatic duct cell carcinogenesis including those associated with the ERK, PKB/AKT, mTOR and STAT3 pathways. The ERK pathway activation appears also increased in duct epithelia adjacent to carcinoma, suggesting tumor micro-environmental effects.

## Background

Pancreatic ductal adenocarcinoma (PDAC) is the most common malignant tumor of the human pancreas. PDAC patients have one of the worst prognoses among all cancer types with a 5-year survival rate of less than 5%. Despite significant advances during the last decade in our molecular knowledge on this disease, the prognosis and management of PDAC patients have remained unchanged [1,2]. The most common genetic aberrations in pancreatic duct cell carcinogenesis involve the activation of *KRAS *oncogene and inactivation of tumor suppressor genes *p16/CDKN2*, *p53 *and *SMAD4/DPC4 *[3]. Less frequently altered genes in PDAC are the amplification of growth factor receptors *EGFR *and *HER2 *[4,5], and the survival signaling transducer *PKBβ/AKT2 *[6]. Additionally, the molecular deregulation of the tyrosine kinase receptor c-MET has been associated with enhanced transcript levels [7]. The protein products of these genes play important regulatory roles in cell proliferation, survival, motility, invasion and differentiation. There is increasing realization that the biochemical activities and cellular functions of these genes constitute part of a complex network of interacting signaling pathways [8]. Activities of these pathways are highly dependent on the reversible phosphorylation of tyrosine, threonine or serine residues of signal transduction proteins.

Despite a significant gain of knowledge on genes that are differentially expressed in PDAC compared with normal pancreas or duct cells, the associated changes in signal transduction networks have not yet been extensively characterized. Studies on the activation of singular pathways by immunohistochemistry (IHC) with phosphorylation-specific antibodies have been reported for PKB/AKT [9,10], p70/S6K [11], NFκB [12,13] and STAT3 [14]. These signaling proteins are potentially major signaling hubs downstream of growth factor receptors that are overexpressed in proportions of PDAC including EGFR (31% to 58%) [5,15,16], HER2 (20%) [4], c-MET/hepatocyte growth factor receptor (78%) [17], c-KIT/stem cell receptor (38%) [18]. However, the IHC analyses performed in these studies rarely included pathway activities in normal pancreatic cellular compartments including the centroacinar, duct or ductular, acinar and islet cells. These components may contain subpopulations of cells that are pancreatic progenitor cells as well as the cell of origin for PDAC [19-21]. The survival of the ductal epithelia has been associated with activated ERK in an inflammatory environment of hereditary pancreatitis, a risk factor for PDAC [22].

To better understand the activation of tyrosine and serine/threonine phosphorylated proteins, we have used IHC analysis to evaluate the levels and activation state of several signaling pathways including the ERK, SRC, STAT3, PTEN/PKB, mTOR/S6K/S6, β-catenin (βCAT) and SMAD4 in PDAC cells and epithelia of normal pancreas.

## Methods

### Tissue material

This study has been approved by the Research Ethics Board of the University Health Network (Toronto, ON, Canada) in compliance with applicable national Tri-Council ethics principles. The formalin-fixed and paraffin embedded (FFPE) samples used in this study were tumor and adjacent normal pancreatic tissue obtained within 30 minutes after Whipple's resection for pancreatic ductal adenocarcinoma (PDAC) or non-PDAC conditions. The FFPE blocks were made from thin cross sections of tissues collected for snap-frozen tumor banking and were initially intended for a histological quality check of the banked tissue. These samples were particularly suitable for this study due to their limited delay between sampling and fixation.

### Tissue microarray (TMA) construction

Histology was reviewed to assure the correct diagnosis of the 26 PDAC specimens and 12 specimens that were considered "normal" pancreas from patients with non-pancreatic conditions (e.g. Ampulla of Vater, bile duct and stomach cancers) [see Additional file 1]. The clinicopathological parameters of individual cases are listed in an additional file [see Additional file 2]. H&E slides were used to guide the selection of representative 1.5 mm diameter cores from paraffin blocks containing viable tumor areas and morphologically normal pancreatic parenchyma. The cores were constructed into a tissue microarray (TMA) using a manual tissue arrayer (Beecher Instruments, Sun Prairie, WI). The final TMA contained 67 of 71 (94.3%) cores, (four cores were lost during processing) and each PDAC case was represented by at least one core [see Additional file 3]. Results are reported on 26 tumor cores (T), 13 cores containing adjacent normal parenchyma and non-neoplastic ducts (Dt), and 12 cores containing parenchyma with non-neoplastic tissue from non-PDAC conditions (D).

### Immunohistochemistry

Antibodies and staining methods are summarized in an additional file [see Additional file 2]. Microwave antigen retrieval was performed for all immunostains except CK7, EGFR and p-S6 which were treated with pepsin digestion. Secondary antibodies (anti-mouse and anti-rabbit) were used as provided by the IDetect Ultra HRP system (ID Labs, London, ON). Goat polyclonal antibody (biotin-conjugated anti-goat IgG, 1:300 dilution), NovaRed peroxidase substrate and hematoxylin counterstain were purchased from Vector Laboratories (Burlingame, CA). A negative control slide was processed with a mix of pooled secondary antibodies, omitting primary antibody incubation [see Additional file 1].

### IHC scoring system

The immunostained slides were scanned using an Aperio CS Scanner (Vista, CA) at 20× magnification. Either slides or digital images were used for scoring by three independent evaluators (NAP, JS, VI) without prior knowledge of the core source or stained target antigen. Final scores were based on a consensus of the three evaluators. A single intensity score was obtained since the intensity of staining within each core was mostly homogeneous. Intensity was scored as 0 for absence of staining, 1 for weak, 2 for moderate, and 3 for strong staining. Scores were recorded for the different cell types as well as their nuclear and cytoplasmic compartments. EGFR and MET receptor were scored for their plasma membrane staining only.

### Statistical analysis

Unsupervised hierarchical clustering using the agglomeration rule average linkage placed cases and antibodies next to each other if they were most similar in their IHC profiles (free software Genesis [23]). The relationship of two categorical variables was calculated using Fisher's exact test. Data of categorical variables were arbitrarily split into low and high groups to maximize statistical power obtained by equalizing the number of samples in each groups. The strength of the relationship between two variables was calculated using Spearman's rank correlation coefficient, Rho (ρ). No adjustment for multiple testing was done. Statistical analyses were performed in SAS Version 9.1 (SAS Institute, Cary, NC).

## Results

### Differential expression of signaling proteins in PDAC

**Figure 1:**
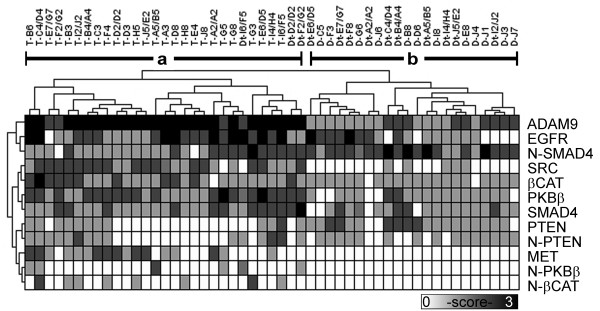
**Signaling proteins in PDAC and ductal epithelia**. Signaling protein levels in the TMA specimens are represented in a grayscale map of IHC intensity levels. Tissue cores are identified as tumor (T), ducts from normal pancreas in PDAC (Dt), or ducts from non-PDAC (D). Array position is designated using x-y coordinates (A-J, 1–8), and specimens derived from the same case have identical nomenclature (T coordinates/Dt coordinates). Protein distribution is cytoplasmic unless designated as nuclear (N-). Common branches show expression similarities in specimens and proteins using an unsupervised hierarchical clustering analysis. The two largest branches contain specimens of tumor (a) and histologically normal ducts (b).

A heat map in Figure 1 shows relative expression levels of eight signaling proteins. The plasma membrane expression of EGFR (p = 0.02) and MET receptor (p < 0.0001) and the cytoplasmic proteins ADAM9 (p < 0.001), SRC (p < 0.0001), PKBβ (p = 0.0005) and βCAT (p = 0.005) were significantly higher in PDAC cells than in histologically normal ducts. In contrast, levels of the two known tumor suppressor genes cytoplasmic PTEN (p = 0.01) and nuclear SMAD4 (p = 0.0005) were significantly lower in PDAC cells than in normal ducts. The p-values of Fisher's exact tests were calculated using proportions of PDAC cells and ducts that expressed high levels of proteins [see Additional file 3]. Unsupervised hierarchical clustering segregated tumor specimens (group a) and normal ducts adjacent to PDAC and non-PDAC pancreas (group b). As expected, cytokeratin 7 was expressed in all PDAC cells and normal ducts (results not shown).

### Cytoplasmic activated protein profiles

**Figure 2:**
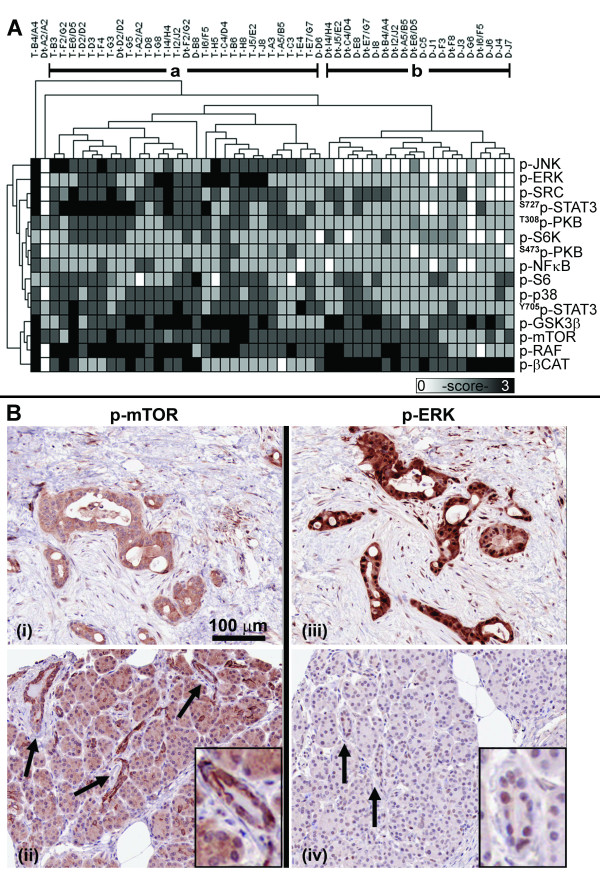
**Cytoplasmic protein activity levels**. (A) Cytoplasmic activated protein profiles are represented by a grayscale map of IHC intensity levels. The two largest branches derived from an unsupervised hierarchical clustering analysis show similarities in tumor specimens (a) and histologically normal ducts (b). (B) Representative IHC images of activated mTOR (i, ii) and ERK (iii, iv) from the TMA of tumor (T-B4/A4, i, iii) and ducts of non-PDAC pancreas (D-J3, ii, iv, arrows). A typical duct is digitally enlarged (insert).

An unsupervised clustering of cytoplasmic activated protein levels shows that tumor specimens (Figure 2A, group a) are more similar to each other than to histologically normal ducts (group b). Only five of twenty five specimens of normal ducts were misclassified as tumor, while none of the tumors were misclassified as normal ducts. The cytoplasmic levels of activated p-ERK (p < 0.001), p-SRC (p = 0.0005), ^T308^p-PKB (p < 0.0001), p-p38 (p = 0.02), and their putative downstream substrates p-JNK (p < 0.0001), ^S727^p-STAT3 (p = 0.005), ^Y705^p-STAT3 (p = 0.0005), p-GSK3β (p = 0.006), p-RAF (p = 0.01) and p-mTOR (p = 0.05) were significantly higher in PDAC cells than in ducts. Degradation-targeted p-βCAT levels significantly decreased (p < 0.0001) in PDAC cells compared with ducts, consistent with the observation of an accumulation of βCAT in PDAC compared with ducts (Figure 1). The four cytoplasmic proteins, ^S473^p-PKB, p-S6K, p-S6 and p-NFκB were similarly expressed in PDAC cells and ducts (Table 1). Although the cytoplasm is the known cellular compartment of activity for the mTOR/S6K/S6 pathway (Figure 2B, i and 2B, ii), a subset of activated signaling proteins are also known to translocate into the nucleus. For example, p-ERK was detected at high levels in the cytoplasm as well as the nuclei of cancer cells (Figure 2B, iii). Low levels of nuclear p-ERK were detected in normal pancreatic ductal cells and acinar (Figure 2B, iv).

**Table 1:** Activated protein levels in PDAC compared with histologically normal duct cells

Marker	High IHC score	PDAC (%)	Duct (%)	p-value
Cytoplasm				
**p-JNK**	**≥ 2**	**77**	**4**	**<0.0001**
**p-ERK**	**≥ 2**	**62**	**8**	**<0.0001**
**p-SRC**	**≥ 2**	**73**	**40**	**0.0005**
^S727^**p-STAT3**	**≥ 3**	**38**	**4**	**0.005**
^T308^**p-PKB**	**≥ 2**	**69**	**12**	**<0.0001**
p-S6K	≥2	27	16	0.5
^S473^p-PKB	≥2	15	0	0.1
p-NFκB	≥2	19	4	0.2
p-S6	≥2	54	40	0.4
**p-p38**	**≥ 2**	**65**	**20**	**0.002**
^Y705^**p-STAT3**	**≥ 2**	**85**	**26**	**0.0005**
**p-GSK3β**	**≥ 3**	**50**	**12**	**0.006**
**p-mTOR**	**≥ 3**	**19**	**0**	**0.05**
**p-RAF**	**≥ 3**	**62**	**24**	**0.01**
**p-βCAT**	**≥ 2**	**42**	**96**	**<0.0001**
				
Nucleus				
p-NFκB	≥3	50	56	1
**p-ERK**	**≥ 3**	**62**	**16**	**0.001**
**p-p38**	**≥ 3**	**62**	**20**	**0.004**
**p-JNK**	**≥ 3**	**26**	**0**	**0.01**
^T308^p-PKB	≥3	27	8	0.1
^S727^p-STAT3	≥2	77	80	1
^S473^**p-PKB**	**≥ 2**	**62**	**16**	**0.001**
^Y705^p-STAT3	≥2	69	56	0.4
p-βCAT	≥2	35	32	1
p-GSK3β	≥1	23	20	1

Proportions of PDAC and duct specimens are split into low and high groups of IHC scores. The results of Fisher's exact test are highlighted for significant differences (p ≤ 0.05) and trends (p = 0.06). Listed are percentages of specimens with high protein levels based on a total of 26 PDAC (T specimens) and 25 normal ducts (D and Dt specimens).

### Nuclear activated protein profiles

**Figure 3:**
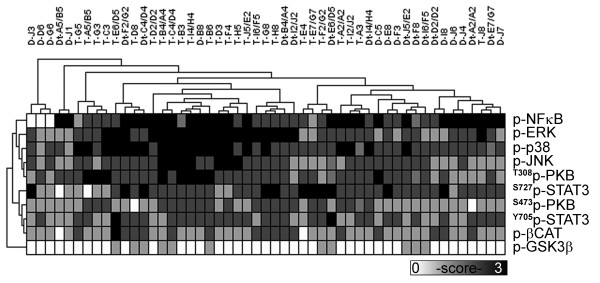
**Nuclear protein activity levels**. Nuclear activated protein profiles are represented by a grayscale map of IHC intensity levels. Common branches show protein level similarities in specimens and proteins from an unsupervised hierarchical clustering analysis.

Since many signaling proteins translocate into the nucleus to serve as transcription factors after being activated by phosphorylation, we explored their profiles in PDAC cells compared with histologically normal ductal epithelia. An unsupervised clustering of the nuclear activated protein levels showed an absence of clustering between tumor and normal ducts (Figure 3). Levels of nuclear activated proteins, including p-NFκB, p-STAT3, p-βCAT, p-GSK3β and ^T308^p-PKB, were similarly expressed in PDAC cells and normal ducts. However, the levels of four proteins, p-ERK (p = 0.001), p-p38 (p = 0.004), p-JNK (p = 0.01), and ^S473^p-PKB (p = 0.001) were significantly higher in nuclei of PDAC cells than in normal ducts (Table 1). Cancer cells also displayed significant moderate to strong relationships between cytoplasmic and nuclear levels for several proteins: p-ERK (ρ = 0.72, p < 0.001), p-p38 (ρ = 0.56, p = 0.004), ^T308^p-PKB (ρ = 0.52, p = 0.007), SMAD4 (ρ = 0.63, p < 0.001) and PTEN (ρ = 0.87, p < 0.001). These associations were absent in ductal epithelia from non-PDAC specimens except for an association of cytoplasmic and nuclear levels for p-p38 (ρ = 0.61, p < 0.001) in the ductal epithelia adjacent to PDAC [see Additional file 4].

### Protein-protein and clinicopathological relationships

Table 2 shows that proportions of PDAC specimens characterized by the ERK pathway activation were associated with all specimens of high p-mTOR levels (100%, p = 0.05), and its downstream substrates p-S6K (100%, p = 0.02), and a proportion of specimens with high p-S6 (86%, p = 0.005). The activation of p-S6K also correlated with high levels of ^S727^p-STAT3 (86%, p = 0.005) and shows a positive trend with high levels of ^T308^p-PKB (100%, 0.06). The activation of p-STAT3 correlated with high levels of PKBβ (59%, p = 0.004) and the presence of SMAD4 (70%, 0.02). An association was observed between high levels of ^T308^p-PKB and p-JNK (80%, p = 0.05). High levels of inactive p-RAF was correlated with PKBβ (82%, p = 0.009), PTEN (91%, p = 0.01) and p-βCAT (91%, p = 0.01). Protein levels were compared with clinicopathological characteristics of tumors. There was no significant association between tumor grade and individual protein levels (p-value > 0.06, Fisher's exact test, results not shown). However, advanced stage (III and IV) showed a trend in association with higher levels of βCAT (p = 0.04), p-NFκB (p = 0.05) and p-GSK3β (p = 0.05). In contrast, high levels of p-STAT3 (p = 0.01) and loss of PTEN (p = 0.01) appeared not to be associated with cancer progression. Table 3 lists the distribution of PDAC stages that showed trends in associations with proteins levels.

**Table 2:** Associations of cytoplasmic protein levels

	Corresponding protein % specimen (number)		
Protein	Low	High	p-value

**High p-ERK**			
p-mTOR	48 (10/21)	100 (5/5)	0.05
p-S6K	47 (9/19)	100 (7/7)	0.02
p-S6	27 (3/11)	86 (12/14)	0.005
**High**^S727^**p-STAT3**			
PKBβ	0 (0/9)	59 (10/17)	0.004
p-S6K	21 (4/19)	86 (6/7)	0.005
SMAD4	19 (3/16)	70 (7/10)	0.02
**High**^T308^**p-PKB**			
p-JNK	33 (2/6)	80 (16/20)	0.05
p-S6K	58 (11/19)	100 (7/7)	0.06
**High p-RAF**			
PKBβ	22 (2/9)	82 (14/17)	0.009
PTEN	40 (6/15)	91 (10/11)	0.01
p-βCAT	40 (6/15)	91 (10/11)	0.01

Listed are high levels of targeted cytoplasmic proteins (highlighted) that significantly associated with either low or high levels for corresponding cytoplasmic proteins (Fisher's exact test, p ≤ 0.05), and trends (p = 0.06).

**Table 3:** Protein levels which characterized advanced PDAC stage

	Number of specimens (%)								
Marker	Low protein level				High protein level				p-value

	I	II	III	IV	I	II	III	IV	

Cytoplasm									
PTEN	1 (7)	8 (53)	6 (40)	0	1 (9)	0	8 (73)	2 (18)	0.01
βCAT	1 (6)	8 (50)	5 (31)	2 (13)	0	1 (10)	9 (90)	0	0.04
p-NFκB	1 (5)	9 (47)	7 (37)	2(11)	0	0	5 (100)	0	0.05
Nucleus									
^Y705^p-STAT3	0	0	6 (75)	2 (25)	1 (6)	9 (50)	8 (44)	0	0.01
p-GSK3β	1 (5)	9 (45)	8 (40)	2 (10)	0	0	6 (100)	0	0.05

Listed are tumors of stage I to IV, and their proportions (%) in low/high protein levels. Advanced PDAC stage (III and IV) associated with high protein levels (PTEN, βCAT, p-NFκB and p-GSK3β) and low p-STAT3 levels (Fisher's exact test, p ≤ 0.05).

### Signaling protein profile in normal pancreatic epithelia

**Figure 4:**
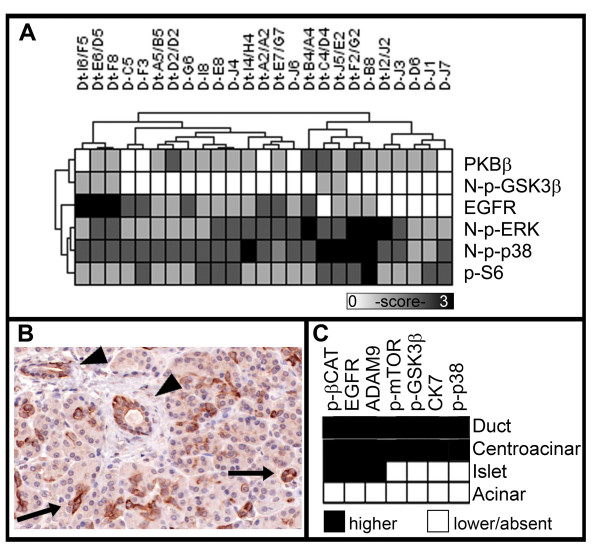
**Ductal epithelia and the pancreas parenchyma**. (A) The profile of six proteins shows significant differences in mean protein levels in ducts of normal pancreas in PDAC compared with ducts in non-PDAC: PKBβ (p = 0.03), nuclear (N-) p-GSK3β (p = 0.008), EGFR (p = 0.055), N-p-ERK (p = 0.04), N-p-p38 (p = 0.03) and p-S6 (p = 0.03). (B) A representative image of centroacinar cells (arrows) and larger ducts (arrowheads) shows positive staining for p-mTOR. (C) A subset of the signaling proteins shows differential staining among the cellular components of the pancreas.

A subset of the signaling proteins examined in this study differentially characterized histologically normal ducts adjacent to PDAC compared with ducts in non-PDAC pancreas (Figure 4A). Mean levels of five proteins, cytoplasmic PKBβ (p = 0.03) and p-S6 (0.03), and nuclear p-GSK3β (p = 0.008), p-ERK (p = 0.04) and p-p38 (p = 0.03), were significantly higher in ducts adjacent to PDAC than in non-PDAC pancreas [see Additional file 5]. EGFR levels showed a tendency (p = 0.055) to be higher in ducts adjacent to PDAC compared with non-PDAC pancreas.

Centroacinar cells, which are the terminal ducts lining the centre of the acini, as expected showed a protein profile that was similar compared with larger ducts (Figure 4B). The seven proteins, CK7, ADAM9, EGFR, p-βCAT, p-GSK3β, p-mTOR and p-p38 were present in both centroacinar and ductal components (Figure 4C). The high expression of p-βCAT, EGFR and ADAM9 characterized islet cells, and relatively lower levels of all seven proteins characterized acinar cells.

## Discussion

**Figure 5:**
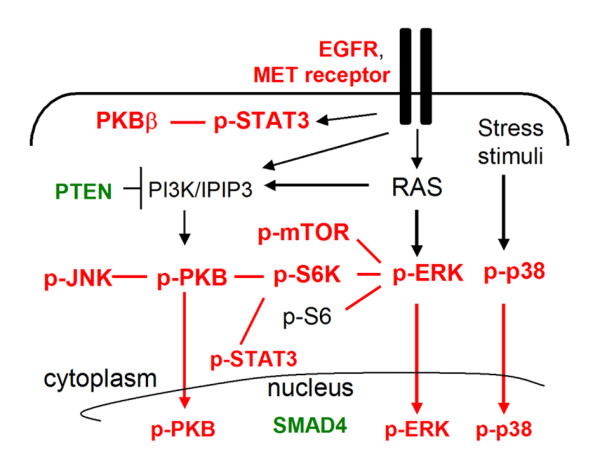
**A schematic of signaling pathways in PDAC**. Higher (red) and lower (green) signaling protein levels characterized tumors compared with non-neoplastic ducts. Relationships between proteins are positive (line), stimulatory (arrows) or inhibitory (T-bar).

In this study, we used immunohistochemistry (IHC) analysis to profile multiple signaling pathways involved in growth and progression of PDAC. To our knowledge, this is the most comprehensive analysis of signaling protein profiles in PDAC cells compared with normal pancreatic duct cells. To confirm the validity of our study, we showed similar frequencies of loss of PTEN and SMAD4 expression in our PDAC samples [24,25], and higher levels of ADAM9 and βCAT in tumor compared with normal ducts as previously reported [1,26]. Our results suggest that higher levels of PTEN (8/9 cases) and lower STAT3 activation (6/6 cases) were associated with advanced PDAC. Our results reveal that the predominantly activated signaling proteins in PDAC include those associated with the ERK, mTOR, STAT3, PKB and p38 pathways (Figure 5).

The trend for associations in (phospho-) protein levels between ERK and the mTOR/S6K/S6 pathway, as well as S6K and PKB or STAT3 suggest a co-activation of these pathways or their crosstalk in PDAC. We propose that further evaluations of ERK and mTOR pathways in PDAC as possible targets for drug combinations are warranted, since single molecule-directed strategies, such as the EGFR inhibitor erlotinib only had a limited effect on patient survival [27].

Our results further suggest that PKB activity lacked significant association with its antagonistic regulator PTEN as well as with downstream PKB substrates including GSK3β, RAF and mTOR. Previous studies reported that high levels of PKBβ (17/61 cases) [10] or loss of PTEN (3/9 cases) were associated with enhanced PKB activity [28] in PDAC. Additionally, the loss of PTEN was found only in a subset of PDAC specimens characterized by enhanced PKBβ activity (2/12 cases) [29]. These inconsistencies may arise from methodological differences (e.g. varying pre-fixation time with impact on phosphorylation states) [30], or the limited number of cases included in previous studies and merit further evaluation.

The levels of a subset of signaling pathways including p-ERK, p-p38, PKBβ or EGFR were enhanced in histologically normal duct cells in PDAC compared with non-PDAC pancreas. This is consistent with a pro-survival role of ERK and JNK in ductal epithelia of a murine hereditary pancreatitis model [22], and of enhanced EGFR expression in chronic pancreatitis [31]. It is conceivable that the activation of these signaling pathways could contribute to early stages of duct cell carcinogenesis or reflect tumor micro-environmental effects of PDAC.

## Conclusion

Our observations suggest that multiple signaling proteins are activated in pancreatic duct cell carcinogenesis including those associated with the ERK, mTOR, STAT3 and PKB pathways. Activation of the ERK pathway also appears increased in duct epithelia adjacent to carcinoma, suggesting a tumor micro-environmental effect.

## Competing interests

The author(s) declare that they have no competing interests.

## Authors' contributions

NAP participated in the study design, evaluated and analyzed the data, and drafted the manuscript. JS participated in the pathological data evaluation and assisted in the manuscript writing. VI reviewed slides for pathological determination of tumor content and assisted in the data evaluation. GP advised and performed statistical analyses. DWH participated in the study design and assessed clinical records. MST conceived of the study, participated in the study design and coordination, and drafting of the manuscript.

## Pre-publication history

The pre-publication history for this paper can be accessed here:



## Supplementary Material

Additional file 1 Additional Figure 1 Tissue microarray. H&E stained TMA and a representative core of negative control staining. Additional Table 1A Summary of Clinical parameters. Identifies surgical procedure, age, sex, tumor stage and grade of patients. Additional Table 1B Clinicopathological parameters of PDAC cases. Sex, age, tumor stage and tumor grade is listed for individual cases. Click here for file

Additional file 2 Additional Table 2 Antibody list. Antibody description, source, method of use and evidence for specificity. Click here for file

Additional file 3 Additional Table 3 Protein levels in PDAC compared with non-neoplastic ductal epithelia. Lists significant associations of high/low protein levels with PDAC specimens compared with duct specimens. Click here for file

Additional file 4 Additional Table 4 Relationships between cytoplasmic and nuclear protein. Lists significant associations in protein levels between cytoplasmic and nuclear compartments. Click here for file

Additional file 5 Additional Table 5 Comparisons of non-neoplastic ducts. Analysis of mean protein levels of non-neoplastic ductal epithelia in peritumoral regions compared with ducts from non-PDAC specimens. Click here for file
