# Supplementary material for: Immunohistochemical analysis of changes in signaling pathway activation downstream of growth factor receptors in pancreatic duct cell carcinogenesis
Source: BMC Cancer. 2008 Feb 6;8:43. doi: 10.1186/1471-2407-8-43 (PMC2270852; doi:10.1186/1471-2407-8-43)
Supplement: Additional file 1 — Additional Figure 1 Tissue microarray. H&E stained TMA and a representative core of negative control staining. Additional Table 1A Summary of Clinical parameters. Identifies surgical procedure, age, sex, tumor stage and grade of patients. Additional Table 1B Clinicopathological parameters of PDAC cases. Sex, age, tumor stage and tumor grade is listed for individual cases. [file 1471-2407-8-43-S1.pdf]

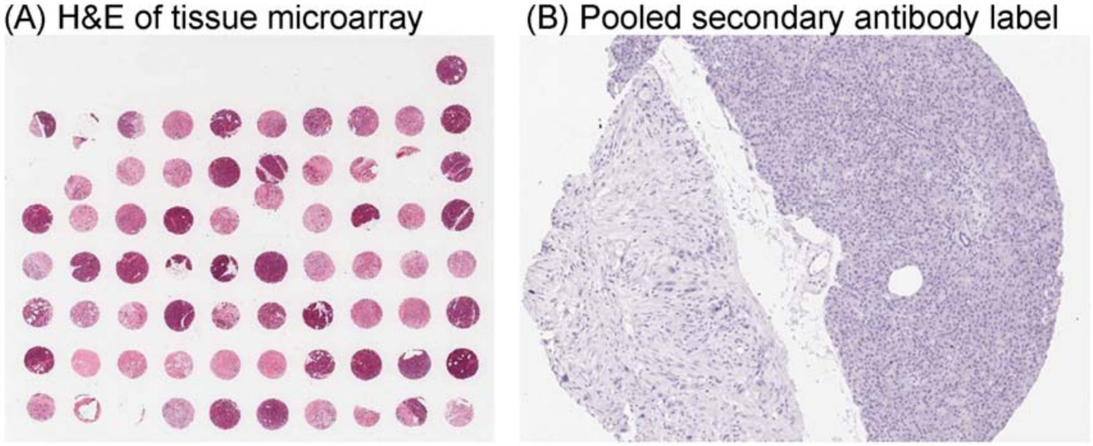

Additional Figure 1 Tissue microarray. (A) An H&E stained TMA slide containing 1.5 mm specimen cores. (B) A representative tissue core shows no staining in the absence of primary antibodies. Images were captured using an Aperio CS scanner.

Additional Table 1A Summary of clinical parameters

Clinical Parameters		
	No. (%)	
	Control*	PDAC
Surgery		
Whipple procedure	12 (100)	22 (85)
Distal pancreatectomy	-	4 (15)
Total	12	26
Age at diagnosis (years)		
Median	67	58
Range	39-76	41-75
Sex		
Female	4 (31)	13 (50)
Male	8 (67)	13 (50)
Stage		
I	-	1 (4)
II	-	9 (34)
III	-	14 (54)
IV	-	2 (8)
Grade		
Well	-	3 (11)
Moderate	-	14 (54)
Poor	-	9 (35)

*Control specimens included one adenoma of the duodenum and jejunum, and five carcinomas of Ampulla of Vater and six carcinomas of bile duct.

Additional Table 1B Clinicopathological parameters of PDAC cases

Tumor case ID	Sex	Age	Tumor stage	Tumor grade
A2/A2	M	65	pT3N1aMx	3
A3	F	68	pT3N1aMx	2
A5/B5	M	55	pT3N0Mx	2
B3	F	54	T2N1	3
B4/A4	F	57	pT1N1aM0	1
B6	F	41	T3N1b	2
C3	M	51	pT4N0Mx	3
C4/D4	F	58	pT3N1bMx	2
D2/D2	M	64	T2N1a	2
D3	M	63	T3N	3
D8	M	62	T3N1bMx	2
E4	F	53	T3N1	2
E6/D5	M	57	T3N1 aMx	2
E7/G7	M	63	T3N0	2
F2/G2	M	63	T2N1	1
F4	M	47	T3N1	2
G3	F	66	pT3N1bM0	2
G5	F	58	pT2N1aMx	1
G8	M	53	T3	3
H5	F	58	pT3N0Mx	3
H8	M	75	T3N0	3
I2/J2	F	54	T3N1	2
I4/H4	M	66	pT3N0Mx	2
I6/F5	F	50	T3N1M1	3
J5/E2	F	68	pT3pN0	3
J8	F	69	T2N0	2
