# Supplementary material for: Immunohistochemical analysis of changes in signaling pathway activation downstream of growth factor receptors in pancreatic duct cell carcinogenesis
Source: BMC Cancer. 2008 Feb 6;8:43. doi: 10.1186/1471-2407-8-43 (PMC2270852; doi:10.1186/1471-2407-8-43)

Additional Table 2 Antibody list

Protein Name	Antibody	Dilution factor	Clone	*Supplier / Cat # / Applications	Antibody performance references
a disintegrin and metalloproteinase domain 9	ADAM9	400	Goat/Pc	R&D Systems / AF949 / IHC	Grutzmann R. Br J Cancer.2004;90(5):1053-8.
βcatenin	βCAT	300	Mc, clone 14	BD / 610153 / IHC	[§]Al-Aynati MM. Clin Cancer Res.2004; 10(4):1235-40
phospho-βcatenin, targeted for degradation (T41/S45)	p-βCAT	50	Rb/Pc	CST / 9565 / IHC	Western blot shown on product datasheet
cytokeratin 7	CK7	2000	Mc, clone OV-TL 12/30	Dako / M7018 / IHC	
epidermal growth factor receptor	EGFR	50	Mc, clone 31G7	Zymed / 28-8763	[§]Agulnik M. J Clin Oncol.2007;25(25):3978-84.
phospho-mitogen activated protein kinase, ERK1/2 (T202/Y204)	p-ERK	100	Rb/Mc, clone 20G11	CST / 4376 / IHC preferred	[§]Agulnik M. J Clin Oncol.2007;25(16):2184-2190.<br>[§§]Western blot
phospho-glycogen synthase kinase 3β (S9), inhibited	p-GSK3β	500	Rb/Pc	CST / 9336 / IHC	[§]Al-Aynati MM. Clin Cancer Res.2004; 10(4):1235-40<br>[§]Pham NA. Pancreas. 2007;35(3):e16-e26.
phospho-c-jun NH2-terminal kinase (T183/Y185)	p-JNK	50	Mc	BD / 612540 / W	Western blot shown on product datasheet
met proto-oncogene / hepatocyte growth factor receptor	MET	200	Mc, clone DL-21	Upstate / 05-238	Kong-Beltran M. Cancer Res.2006;66:283-289.
nuclear factor-κB/ p65, phosphorylated (S276)	p-NFκB	100	Rb/Pc	CST / 3037 / IHC	[§]Agulnik M. J Clin Oncol.2007;25(16):2184-2190.<br>[§]Schwock J. Diagn Mol Pathol.2007;16(3):130-140.
phospho-p38 mitogen activated protein kinase (T180/Y182)	p-p38	200	Rb/Mc	CST / 4631 / IHC specific	[§§]Western blot
protein kinase B β/Akt2	PKBβ	300	Rb/Mc	CST / 4057 / IHC specific	[§]Pham NA. Pancreas. 2007;35(3):e16-e26.
phospho-protein kinase B / Akt	^{S473}p-PKB	100	Rb/Mc	CST/ 3787 / IHC specific	[§§]Western blot
	^{T308}p-PKB	200	Rb/Pc	CST / 9275 / W	[§]Paraffin cell blocks, serum starved/ stimulated, in-house.<br>[§]Pham NA. Pancreas. 2007;35(3):e16-e26.
phosphatase and tensin homolog	PTEN	500	Rb/Mc	CST / 9559 / IHC	[§]Duran I. Br J Cancer.2006;95(9):1148-

					54.
phospho-cRaf-1 (S259), inhibited	p-RAF	200	Rb/Pc	CST / 9421 / W	Western blot shown on product datasheet
phospho-mammalian target of rapamycin (S2448)	p-mTOR	400	Rb/Pc	CST / 2971 / W	§Duran I. Br J Cancer.2006;95(9):1148-54.
phospho-ribosomal protein p70/S6 kinase (T389)	p-S6K	100	M, clone 1A5	CST / 9206	Riemenschneider MJ. Cancer Res.2006; 66(11):5618-23. §Yau CYF. Can Res.2005;65(4):1497-1504.
phospho-ribosomal protein S6 (S235/236)	p-S6	200	Rb/Pc	CST / 2211 / IHC	§Duran I. Br J Cancer.2006;95(9):1148-54. §Birle DC. Mol Cancer Ther.2006;5(10):2494-502.
mothers against decapentaplegic homolog 4	SMAD4	100	M	SC / sc-7966 / IHC	§Qian J. Can Res.2005;65(12):5045-5053.
v-src sarcoma viral oncogene homolog	SRC	25	M	Upstate / 05-184	§§Western blot
phospho-src (Y416/Y419), autophosphorylated site	p-SRC	50	Rb/Pc	CST / 2101	§§Western blot
phospho-signal transducer and activator of transcription 3	^{S727}p-STAT3	200	M	BD / 612542 / IF	§Pham NA. Diagn Pathol.2007;2:8-17.
	^{Y705}p-STAT3	100		BD / 612356 / IC	§Schwock J. Diagn Mol Pathol.2007;16(3):130-140.

Abbreviations: Rb, rabbit; M, mouse; Pc, polyclonal; Mc, monoclonal.

*Supplier: R&D Systems, Minneapolis, MN; Cell Signaling Technology (CST), Danvers, MA; Dako, Mississauga ON; BD Biosciences (BD), Mississauga, ON; Santa Cruz Biotechnology (SC), Santa Cruz, CA; Upstate, Charlottesville, VA and Zymed Laboratories, San Francisco, CA. Applications: supplier tested applications include immunohistochemistry, IHC; immunofluorescence, IF; immunocytochemistry, IC and Western blotting, W. §Performance of the antibodies have been tested in our laboratory for IHC, IF and Western blotting on samples derived from clinical specimens, tumor xenografts and cell cultures. §§Western blots are shown below from data in our laboratory not yet published.

§§ **(Additional Table 2) Western blots from data in our laboratory not yet published. Arrow indicates detection of specific protein band.**

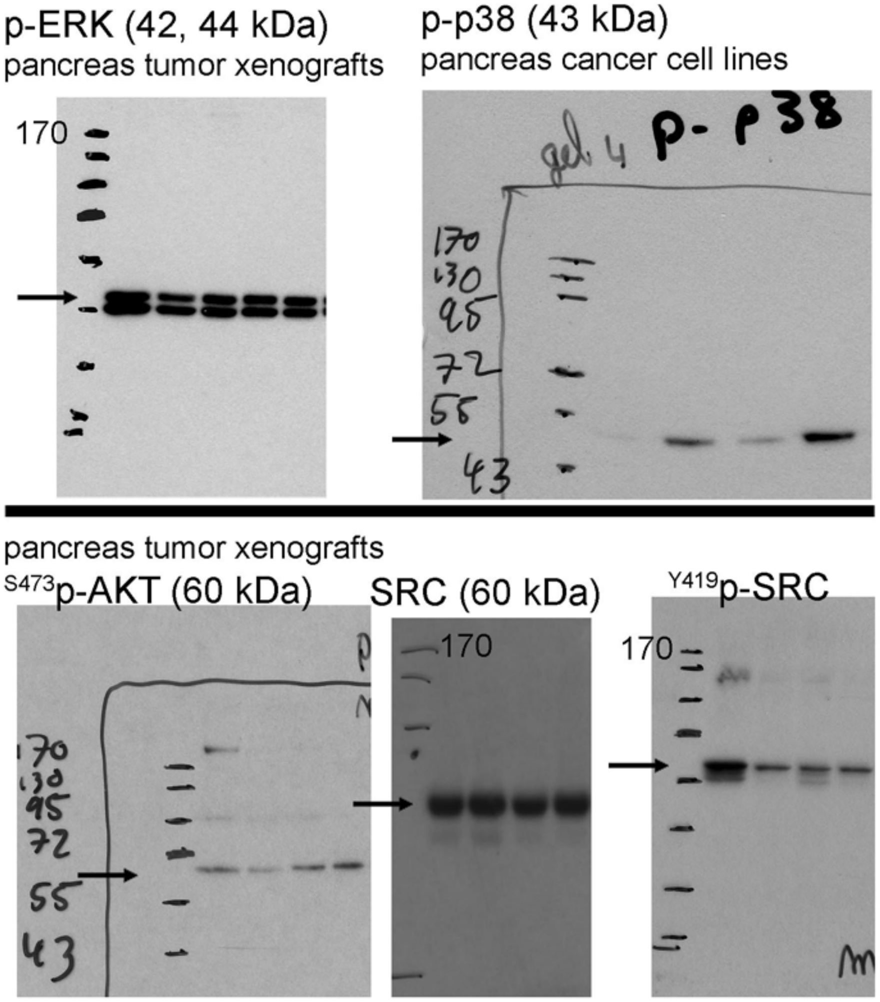

Supplement: Additional file 2 — Additional Table 2 Antibody list. Antibody description, source, method of use and evidence for specificity. [file 1471-2407-8-43-S2.pdf]
