# Supplementary material for: Immunohistochemical analysis of changes in signaling pathway activation downstream of growth factor receptors in pancreatic duct cell carcinogenesis
Source: BMC Cancer. 2008 Feb 6;8:43. doi: 10.1186/1471-2407-8-43 (PMC2270852; doi:10.1186/1471-2407-8-43)
Supplement: Additional file 3 — Additional Table 3 Protein levels in PDAC compared with non-neoplastic ductal epithelia. Lists significant associations of high/low protein levels with PDAC specimens compared with duct specimens. [file 1471-2407-8-43-S3.pdf]

Additional Table 3 Protein levels in PDAC compared to non-neoplastic ductal epithelia

Marker	High IHC score	PDAC (%)	Duct (%)	p-value
Plasma membrane				
**EGFR**	**≥2**	**69**	**36**	**0.02**
**MET**	**≥1**	**65**	**0**	**<0.0001**
Cytoplasm				
**ADAM9**	**≥3**	**88**	**8**	**<0.001**
**SRC**	**≥2**	**58**	**4**	**<0.0001**
**βCAT**	**≥2**	**38**	**4**	**0.005**
**PKBβ**	**≥2**	**65**	**16**	**0.0005**
SMAD4	≥2	38	24	0.4
**PTEN**	**≥1**	**42**	**80**	**0.01**
Nucleus				
**SMAD4**	**≥2**	**35**	**84**	**0.0005**
PTEN	≥1	50	68	0.2
PKBβ	≥1	15	4	0.3
**βCAT**	**≥1**	**31**	**0**	**0.004**

Listed are percentages of specimens with high levels of proteins based on a total of 26 PDAC and 25 non-neoplastic specimens. Highlighted are significant differences in proportions of PDAC compared to ducts in low and high protein expression levels using Fisher's exact test ($p \leq 0.05$).
