# Supplementary material for: Immunohistochemical analysis of changes in signaling pathway activation downstream of growth factor receptors in pancreatic duct cell carcinogenesis
Source: BMC Cancer. 2008 Feb 6;8:43. doi: 10.1186/1471-2407-8-43 (PMC2270852; doi:10.1186/1471-2407-8-43)
Supplement: Additional file 4 — Additional Table 4 Relationships between cytoplasmic and nuclear protein. Lists significant associations in protein levels between cytoplasmic and nuclear compartments. [file 1471-2407-8-43-S4.pdf]

Additional Table 4 Relationships between cytoplasmic and nuclear protein

	Combined cell types		PDAC		Ductal epithelia adjacent to PDAC		Duct cell in non-PDAC specimen	
Protein	ρ	p-value	ρ	p-value	ρ	p-value	ρ	p-value
S727p-STAT3	0.02	0.91	-0.22	0.29	0.10	0.73	0.35	0.087
βCAT	0.21	0.13	0.09	0.67	-	-	-	-
Y705STAT3	0.37	0.008	0.22	0.28	0.19	0.53	0.43	0.031
p-βCAT	0.07	0.62	0.25	0.21	-0.05	0.86	0.05	0.81
p-GSK3β	0.19	0.18	0.28	0.17	0.02	0.94	0.08	0.72
S473p-PKB	0.43	0.002	0.32	0.11	0.49	0.088	0.37	0.071
PKBβ	0.39	0.005	0.36	0.072	0.40	0.20	0.35	0.094
p-JNK	0.37	0.009	0.43	0.029	0.03	0.92	-0.05	0.83
p-NFκB	0.41	0.004	0.44	0.029	0.41	0.18	0.41	0.044
**T308p-PKB**	0.51	<0.001	**0.52**	**0.007**	-0.24	0.48	0.24	0.27
**p-p38**	0.63	<0.001	**0.56**	**0.004**	**0.61**	**0.036**	0.44	0.030
**SMAD4**	0.27	0.06	**0.63**	**<0.001**	0.00	1.00	0.22	0.31
**p-ERK**	0.72	<0.001	**0.72**	**<0.001**	0.29	0.34	0.42	0.035
**PTEN**	0.56	<0.001	**0.87**	**<0.001**	0.23	0.46	0.17	0.43

Significant moderate to strong relationships are highlighted (ρ≥0.5, p≤0.05). Identical protein values between the sub-cellular compartments were omitted from the Spearman's test (—).
