# Supplementary material for: Immunohistochemical analysis of changes in signaling pathway activation downstream of growth factor receptors in pancreatic duct cell carcinogenesis
Source: BMC Cancer. 2008 Feb 6;8:43. doi: 10.1186/1471-2407-8-43 (PMC2270852; doi:10.1186/1471-2407-8-43)
Supplement: Additional file 5 — Additional Table 5 Comparisons of non-neoplastic ducts. Analysis of mean protein levels of non-neoplastic ductal epithelia in peritumoral regions compared with ducts from non-PDAC specimens. [file 1471-2407-8-43-S5.pdf]

Additional Table 5 Comparisons of non-neoplastic ducts

Protein	mean level (Dt)	mean level (D)	p-value
ADAM9	1.692	1.417	0.150
**PKBβ-C**	**1.154**	**0.667**	**0.028**
PKBβ-N	0.083	0.000	0.164
p-PKB (S473)-C	0.846	0.917	0.303
p-PKB (S473)-N	1.154	1.083	0.349
p-PKB (T308)-C	1.182	1.000	0.154
p-PKB (T308)-N	1.727	1.750	0.466
p-βCAT-C	2.385	2.667	0.167
p βCAT-N	1.385	1.167	0.163
βCAT –C	1.000	0.917	0.282
βCAT-N	0.000	0.000	—
**EGFR**	**1.538**	**0.917**	**0.055**
p-GSK3β-C	2.154	2.000	0.298
**p-GSK3β-N**	**0.385**	**0.000**	**0.008**
MET	0.000	0.000	—
p-mTOR	1.846	1.667	0.157
p-NFκB-C	1.000	0.750	0.089
p-NFκB-N	2.583	2.083	0.116
p-JNK-C	0.333	0.333	0.500
p-JNK-N	1.583	1.583	0.500
p-ERK-C	1.000	0.750	0.122
**p-ERK-N**	**2.000**	**1.500**	**0.042**
p-p38-C	1.083	1.250	0.204
**p-p38-N**	**2.333**	**1.917**	**0.028**
**p-S6-C**	**1.231**	**1.667**	**0.030**
PTEN-C	1.000	1.083	0.383
PTEN-N	0.538	0.833	0.062
p-RAF-C	2.077	1.500	0.058
p-S6K-C	1.083	0.667	0.092
SMAD4-C	1.308	0.909	0.098
SMAD4-N	2.000	2.000	0.500
SRC-C	0.462	0.167	0.096
p-SRC-C	1.385	1.000	0.108
p-STAT (S727)-C	1.308	0.917	0.092
p-STAT (S727)-N	2.077	1.917	0.305
p-STAT (Y705)-C	1.385	1.333	0.400
p-STAT (Y705)-N	1.692	1.500	0.208

Mean protein levels of non-neoplastic duct epithelia in peritumoral regions (Dt) are compared to ducts from non-cancerous pancreas specimens (D) using Student’s t-test. Protein levels are scored in the cytoplasmic (-C) and nuclear (-N) cellular compartments.
